# Comparative effects of platelets and plasma-derived mitochondria in early-phase Achilles tendon inflammation

**DOI:** 10.1093/rb/rbag127

**Published:** 2026-06-13

**Authors:** Seong-Hoon Kim, Eun-Seo Back, Mina Lim, Mi Jin Kim, Chang-Koo Yun, Yong-Soo Choi

**Affiliations:** Department of Bio-Convergence Science, Graduate School, CHA University, Seongnam 13488, Republic of Korea; Department of Life Sciences, Graduate School, CHA University, Seongnam 13488, Republic of Korea; Department of Bio-Convergence Science, Graduate School, CHA University, Seongnam 13488, Republic of Korea; Department of Life Sciences, CHA University, Seongnam 13488, Republic of Korea; Department of Life Sciences, CHA University, Seongnam 13488, Republic of Korea; The Institute of AI-driven Industrial Biotechnology, Inha University, Incheon 22212, Republic of Korea; Department of Bio-Convergence Science, Graduate School, CHA University, Seongnam 13488, Republic of Korea; Department of Life Sciences, Graduate School, CHA University, Seongnam 13488, Republic of Korea; Department of Life Sciences, CHA University, Seongnam 13488, Republic of Korea; Department of Medicinal Biosciences and Bioengineering, Inha University, Incheon 22212, Republic of Korea

**Keywords:** Achilles tendon injury, plasma-derived mitochondria, platelet-rich plasma, macrophage polarization, early inflammatory response

## Abstract

Tendon repair often results in a scar-like extracellular matrix rather than the restoration of native architecture. Early inflammatory remodeling may be a critical determinant of outcome, and monocyte/macrophage-associated programs are prominent during this window. Motivated by prior *in vitro* observations that platelets and plasma-derived mitochondria (p-mito) are associated with divergent macrophage signatures, we tested their effects in a collagenase-induced rat Achilles tendon injury model. Rats received intratendinous injections of platelets or p-mito on Day 3 post-induction and were analyzed at a Day 10 endpoint. Platelet treatment was associated with an additional transient increase in tendon thickness, whereas p-mito were associated with a sustained post-treatment decline approaching saline-injected sham levels by later assessments. At Day 10, p-mito were associated with the lower operational M1/M2 ratio, lower TNF-α and IL-6 with higher IL-10, and a tendon protein profile characterized by higher type I collagen (COL1) with lower tenascin C relative to platelets. Together, these findings indicate that p-mito are associated with distinct inflammatory and tendon-marker profiles relative to a platelet preparation. These observations suggest that p-mito may represent a mechanistically distinct acellular alternative to the platelet-based preparation used here, with the potential to limit transient inflammatory flare.

## Graphical Abstract

**Graphical Abstract:**
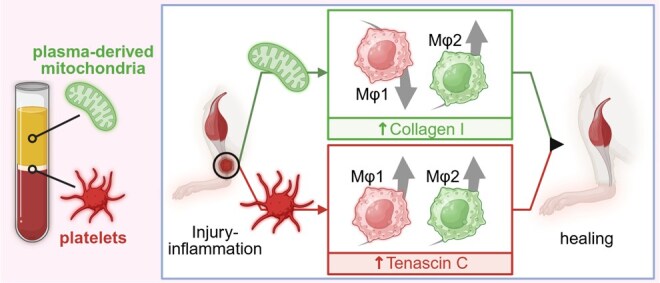

## Introduction

Tendon injury is common and often heals with scar-like extracellular matrix rather than the restoration of native architecture [1, 2]. This can leave persistent pain, reduced mechanical function and a risk of reinjury [3, 4]. The earliest inflammatory phase may be a critical determinant of outcome, yet mechanistic insight is limited because early human specimens are difficult to obtain [5–7]. Innate immune responses are increasingly recognized as contributors to repair, and monocytes and macrophages integrate injury signals with cues that govern remodeling [8, 9]. Although activation states are heterogeneous, an operational distinction between pro-inflammatory ‘M1-like’ programs and pro-resolving ‘M2-like’ programs has been useful [10, 11]. Excessive or prolonged M1-like signaling may delay resolution and impair matrix remodeling [12, 13]. Timely engagement of M2-like programs may support inflammatory resolution and matrix organization [8, 12, 14]. How these programs reorganize during the early stage after tendon injury, and whether they can be guided therapeutically, remain unclear.

**Figure 1:**
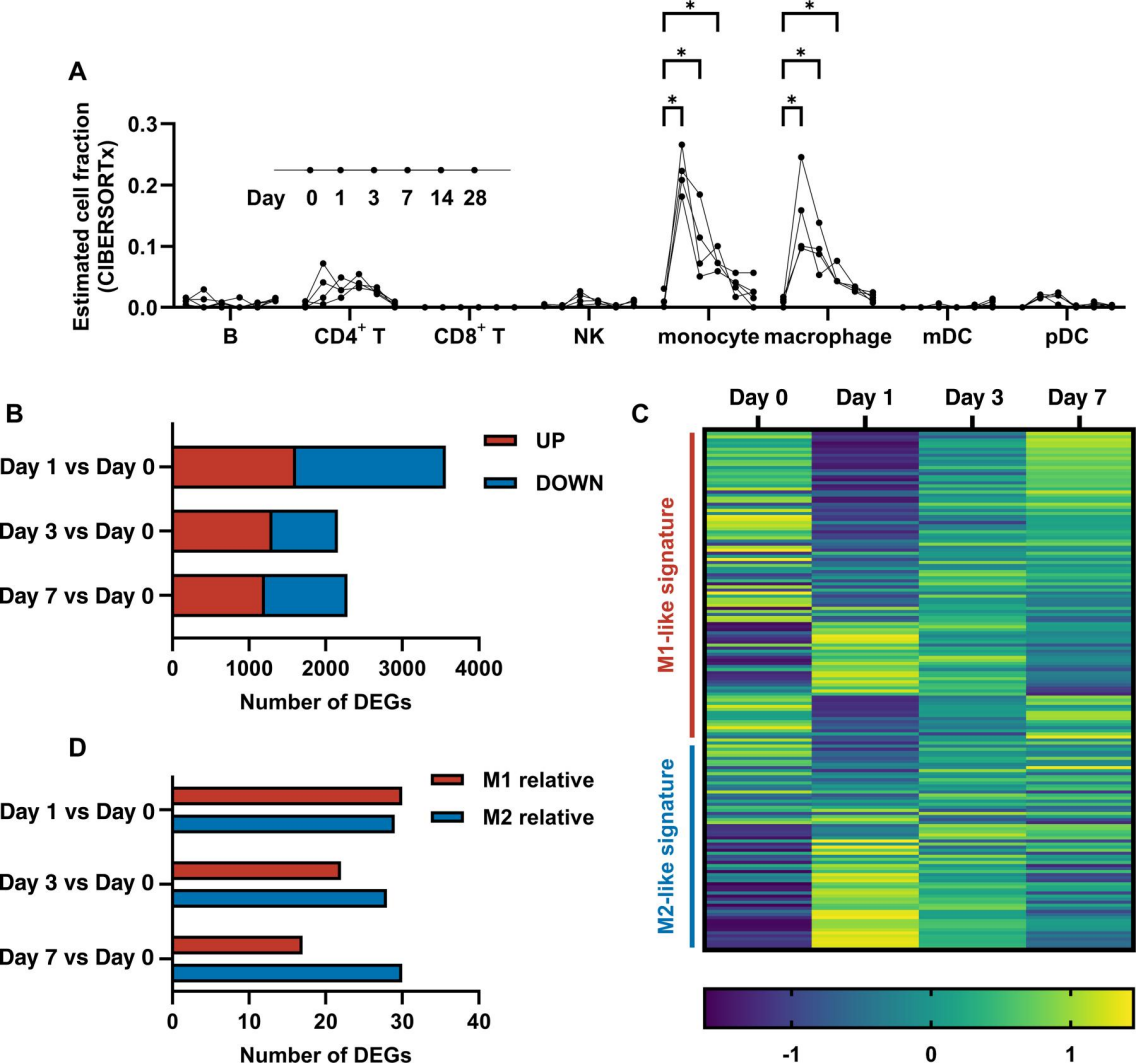
Immune cell dynamics and macrophage polarization signatures during early-stage Achilles tendon injury (GSE288444). (**A**) Relative abundance of immune cell populations inferred from bulk RNA-seq using CIBERSORTx across the indicated time points after puncture-induced tendon injury. Each dot represents an individual sample. Statistical significance was assessed by ordinary two-way ANOVA followed by Dunnett’s multiple-comparisons test (vs Day 0) within each cell type. (**B**) Numbers of differentially expressed genes (DEGs) identified from bulk RNA-seq at Day 1 vs Day 0 (D1vsD0), Day 3 vs Day 0 (D3vsD0) and Day 7 vs Day 0 (D7vsD0), separated into upregulated and downregulated genes (Adj.*P* < 0.05 and |Log_2_FC| > 1). (**C**) Heatmap of per-gene RNA-seq expression *z*-scores calculated across the Days 0, 1, 3 and 7 mean expression values for macrophage polarization gene sets derived from GSEA immunologic signatures: COATES_MACROPHAGE_M1_VS_M2_UP (M1-like signature) and COATES_MACROPHAGE_M1_VS_M2_DN (M2-like signature). (**D**) Numbers of DEGs overlapping the M1-like signature or M2-like signature gene lists in each comparison (D1vsD0, D3vsD0 and D7vsD0), stratified by direction (up/down) using the same thresholds as in (B).

To further support a focus on early myeloid dynamics, we reanalyzed a publicly available multi-time point bulk RNA-seq tendon injury dataset (GSE288444). Immune deconvolution with CIBERSORTx suggested temporal variation in estimated monocyte/macrophage-associated fractions during the first week after injury (Figure 1A) [15–17]. Differential expression analysis further indicated broad transcriptional remodeling across Days 1, 3 and 7 relative to baseline (Figure 1B). When curated macrophage polarization signatures were examined, the heatmap showed time-dependent shifts in genes associated with M1-like and M2-like programs (Figure 1C), and overlap analysis of differentially expressed genes with these signature sets was consistent with dynamic reorganization of macrophage-associated transcriptional programs over Days 0–7 (Figure 1D). Together, these analyses provide descriptive support for focusing on early macrophage-associated inflammatory remodeling, while acknowledging the limitations of bulk profiling and computational deconvolution [18].

Platelet-rich plasma (PRP) is widely used to promote musculoskeletal repair, but it can be associated with transient inflammation and variable clinical response [19–21]. In the present study, however, the *in vivo* comparator was not a clinically standardized PRP formulation but a platelet-based preparation derived during PRP processing and subsequently pelleted and resuspended in normal saline prior to injection. In our prior *in vitro* work, platelets favored M1-like profiles, whereas plasma-derived mitochondria (p-mito), isolated from circulating plasma by simple centrifugation, were associated with more M2-like signatures [22]. These observations led us to hypothesize that p-mito could temper excessive inflammatory priming while supporting macrophage programs linked to inflammatory resolution and early repair [22].

Here, we tested whether these immunomodulatory tendencies translate to an inflamed tendon injury microenvironment *in vivo* and whether p-mito yields responses distinct from platelets under comparable conditions. We used a collagenase-induced Achilles tendon injury model in rats and administered platelets or p-mito during the early inflammatory phase (Day 3 post-induction). Because this model reflects an acute inflammatory injury rather than chronic tendinopathy, we focused on inflammatory resolution and early repair [23–26]. Our objective was to determine whether p-mito more effectively supports macrophage-associated resolution and early repair outcomes than platelets under matched conditions.

## Materials and methods

### Public bulk RNA-seq reanalysis and immune deconvolution (GSE288444)

Bulk RNA-seq data from a puncture-induced Achilles tendon injury time course (GSE288444) were downloaded from the Gene Expression Omnibus (GEO). Raw counts were normalized by library size to counts per million (CPM), and expression values were computed as log_2_(CPM + 1) (pseudocount = 1). Immune cell fractions were inferred using CIBERSORTx with the DerM22 signature matrix and quantile normalization disabled, and mononuclear immune cell fractions were plotted across Days 0–28.

Differential expression was assessed for Day 1 vs Day 0, Day 3 vs Day 0 and Day 7 vs Day 0 using two-sided *t*-tests on log_2_(CPM + 1) values, with *P* value adjusted by the Benjamini–Hochberg procedure. For macrophage polarization signatures, MSigDB gene sets COATES_MACROPHAGE_M1_VS_M2_UP (M1-like) and COATES_MACROPHAGE_M1_VS_M2_DN (M2-like) were used. For heatmap visualization, the mean expression value for each gene at Days 0, 1, 3 and 7 was converted to a per-gene *z*-score across these time points, and the resulting values were visualized as a heatmap.

### Rat collagenase-induced Achilles tendon inflammation model

Male Sprague–Dawley rats (5 weeks old, 200–300 g) were purchased from JABIO. On Day 0, Achilles tendon inflammation was induced by bilateral intratendinous injection of collagenase (0.6 mg in 30 μL normal saline per tendon; Sigma-Aldrich, C0130) after surgical exposure of the tendon under inhalation anesthesia with isoflurane. Injections were delivered into the mid-substance region of the Achilles tendon. Sham animals received bilateral normal saline injections (30 μL per tendon) on Day 0.

On Day 3 post-induction, collagenase-treated animals were allocated to the vehicle, platelet or p-mito groups based on Day 3 tendon thickness to balance baseline swelling across groups (*N* = 3 rats/group). Animals then received bilateral intratendinous injections of platelets (2.5 × 10^7^ in 30 μL per tendon), p-mito (25 μg in 30 μL per tendon) or vehicle (normal saline; 30 μL per tendon). Sham animals received normal saline on Day 3. All injections were performed using an insulin syringe. Animals were followed to a Day 10 terminal endpoint for downstream tissue analyses.

All animal procedures were approved by the Institutional Animal Care and Use Committee of CHA University (IACUC230145).

### Platelets and p-mito preparation

Peripheral blood was obtained from healthy adult volunteers at CHA University Hospital under written informed consent. Blood was collected into EDTA tubes and processed fresh for each experiment. Study procedures were approved by the Institutional Review Board of CHA University (IRB No. 2021-06-044).

For the platelet preparation used for *in vivo* injection, whole blood was centrifuged at 1000 × *g* for 20 min to obtain the PRP fraction. The PRP fraction was centrifuged at 300 × *g* for 5 min, and the supernatant was subsequently centrifuged at 2000 × *g* for 5 min to pellet platelets. All centrifugation steps used for platelet isolation were performed at room temperature to minimize platelet/PRP aggregation during preparation [27, 28]. After final isolation, the platelet pellet was resuspended in normal saline and kept at 4°C until use. Platelet number was determined using a hemocytometer, and suspensions were adjusted to deliver 2.5 × 10^7^ platelets in 30 μL per tendon.

For p-mito, plasma was isolated by centrifugation of whole blood at 1000 × *g* for 20 min. Plasma was centrifuged at 2000 × *g* for 5 min, and the resulting supernatant was centrifuged at 20 000 × *g* for 5 min to pellet p-mito. The pellet was washed once by resuspension in normal saline followed by centrifugation at 20 000 × *g* for 5 min, then resuspended in normal saline for immediate use. The p-mito preparation used in this study followed our previously validated p-mito isolation workflow, in which purity, structural integrity and previously characterized bioenergetic function were characterized by mitochondrial marker enrichment, reduced non-mitochondrial contamination and mitochondrial functional assays [22, 29]. The p-mito dose was quantified by BCA protein assay and adjusted to deliver 25 μg in 30 μL per tendon. The platelet and p-mito preparations were not matched by total protein content, because the platelet preparation consisted of intact platelets quantified by cell number, whereas the p-mito preparation was an isolated mitochondrial fraction quantified by protein mass. The 25 μg p-mito dose was selected based on an empirically derived platelet-associated mitochondrial yield estimate from our preparation workflow [22].

### Serial tendon thickness measurement and gross morphology assessment

Achilles tendon thickness was measured serially in the sham, vehicle, platelet and p-mito groups (*N* = 3 rats/group). At each time point, rats were anesthetized with inhalation anesthesia, the skin overlying the Achilles tendon was incised to expose the tendon, and thickness was measured at the mid-substance using a digital caliper. For each tendon at each time point, thickness was recorded three times and averaged. Left and right Achilles tendon values were then averaged to yield one value per animal per time point. Thickness measurements were performed blinded to group allocation. Between longitudinal measurement time points, the skin incision was closed and secured using skin tape, including wrapping over the incision region.

At the Day 10 endpoint, animals were euthanized by CO_2_ inhalation. For gross morphology assessment, the skin was removed to fully expose the Achilles tendons, and representative images were acquired in dorsal and lateral views.

### Tendon cell dissociation

At the Day 10 endpoint, Achilles tendons were harvested and processed into single-cell suspensions. The left Achilles tendon was used for dissociation. Tendon tissue was finely minced and enzymatically digested in DMEM containing collagenase type II (100 U/mL; Gibco, 17101-015) and DNase I (0.1 mg/mL; STEMCELL Technologies, 07900) for 4 h at 37°C with shaking. Digested material was passed through a 100-µm cell strainer to remove undigested debris. Red blood cells were removed using RBC lysis buffer (BioLegend, 420301). An aliquot of the resulting cells was reserved for flow cytometry, and the remaining cells were used for primary culture.

### Flow cytometry staining, acquisition and analysis of macrophage subsets

Single-cell suspensions generated from Day 10 Achilles tendon digests were used for flow cytometry. Cells were stained in FACS buffer (DPBS supplemented with 1% FBS). All staining steps were performed at 4°C, with antibody incubations for 30 min.

Leukocytes were identified using CD45-APC (Thermo Fisher Scientific, 17-0461-82). Macrophages were identified by CD68 staining using CD68-FITC (MA5-28262) following fixation/permeabilization (Fixation/Permeabilization Kit, BD Biosciences, 554714). CD86 and CD206 were assessed in separate tubes using two panels (CD45/CD68/CD86 or CD45/CD68/CD206). CD86 (BS-1035R) and CD206 (BS-4727R) primary antibodies were detected using a PE-conjugated secondary antibody (12-4739-81). Data were acquired on a CytoFLEX flow cytometer (Beckman Coulter) and analyzed using CytExpert software (v2.4.0).

### Macrophage differentiation, platelet or p-mito exposure and conditioned media collection

Primary rat bone marrow-derived macrophages (BMDMs) were generated from femoral bone marrow of male Sprague–Dawley rats. Femurs were aseptically excised, marrow was flushed with DMEM, and cells were pelleted (300 × *g*, 5 min). Bone marrow cells were seeded in 24-well plates (5 × 10^5^ cells/mL) in DMEM containing 10% FBS and macrophage colony-stimulating factor (10 ng/mL; Gibco, 300-25). Cultures were maintained with medium changes every 2 − 3 days. On Day 7 of differentiation, adherent BMDMs were washed and used for platelet or p-mito exposure experiments.

THP-1 cells (ATCC, TIB-202) were maintained in RPMI-1640 supplemented with 10% FBS (Thermo Fisher Scientific) and 0.05 mM 2-mercaptoethanol at 37°C in a humidified incubator with 5% CO_2_. For macrophage differentiation, THP-1 cells were seeded in 24-well plates (5 × 10^5^ cells/mL) and allowed to adhere for 24 h. Phorbol 12-myristate 13-acetate (Sigma-Aldrich, 79346) was added to a final concentration of 25 nM, and cells were incubated for 48 h.

Differentiated BMDMs and THP-1 macrophages were exposed for 48 h to either platelets (5 × 10^6^ platelets/well) or p-mito (5 μg protein/well). After 48 h exposure to platelets or p-mito, culture supernatants were collected and clarified by centrifugation at 12 000 × *g* for 5 min to remove cells and debris. Clarified supernatants were used as macrophage-conditioned media (CM). When storage was required, CM was aliquoted and stored at −80°C.

### Rat tendon-derived cell and human tenocyte culture and macrophage CM treatment

At the Day 10 endpoint, the left Achilles tendon was harvested and enzymatically dissociated as described above. Cells were resuspended in DMEM supplemented with 10% FBS and maintained at 37°C in a humidified incubator with 5% CO_2_, with medium changes every 2–3 days.

Primary human tenocytes were obtained as a commercial preparation (TEN-F; Zen-Bio) and cultured in DMEM supplemented with 10% FBS at 37°C in a humidified incubator with 5% CO_2_.

Macrophage CM was applied to rat tendon-derived cells or human tenocytes at a final concentration of 10% (v/v) in complete culture medium. Cells were treated when cultures were at approximately 70–80% confluent and exposed to CM for 48 h prior to downstream analyses.

To test whether platelet-CM-associated tenocyte protein changes were reversible, a CM switch paradigm was performed in human tenocytes using THP-1 macrophage CM. Cells were treated with 10% (v/v) platelet-CM for 24 h, washed with DPBS, and then switched to 10% (v/v) p-mito-CM for an additional 24 h (‘switch’). This was compared with a sustained exposure condition in which cells received 10% (v/v) platelet-CM for 48 h (‘sustain’). After the 48 h total exposure, cells were harvested for immunoblot analyses.

### Cytokine ELISA and immunoblotting

At the Day 10 endpoint, the right Achilles tendon was harvested, minced and divided for ELISA and immunoblot analyses.

For ELISA, tendon tissue was homogenized in PBS containing a protease inhibitor cocktail (Quartett, QTPPI1015) at 4°C and clarified by centrifugation (12 000 × *g*, 10 min). TNF-α (RTA00), IL-6 (R6000B) and IL-10 (R1000) were measured using an ELISA kit (R&D Systems), and values were normalized to total protein. The same ELISA kits were used to quantify TNF-α, IL-6 and IL-10 in rat BMDM CM. For THP-1 macrophage CM, TNF-α (Abcam, ab181421), IL-6 (Invitrogen, 88-7066-88), IL-1β (88-7261-88) and IL-10 (R&D Systems, D1000B) were measured.

For immunoblotting, rat tendon tissue, rat tendon-derived cells and human tenocytes were lysed in RIPA buffer (BioSolution, BR002) supplemented with protease inhibitor, clarified by centrifugation (12 000 × *g*, 10 min) and quantified by BCA. The RIPA-soluble supernatant was used for tenomodulin (TNMD), scleraxis (SCX), tenascin C (TNC), matrix metalloproteinase-1 (MMP-1) and β-actin immunoblotting. The insoluble pellet was resuspended in PBS, quantified by BCA, mixed with SDS-PAGE loading buffer containing 5% 2-mercaptoethanol, heated at 60°C for 30 min and centrifuged (12 000 × *g*, 10 min). The resulting supernatant was used for type I collagen (COL1) immunoblotting.

Equal amounts of protein were separated by SDS-PAGE, transferred to PVDF membranes using a wet transfer system, blocked with 5% BSA in TBST for 1 h at room temperature and incubated overnight at 4°C with primary antibodies. Membranes were washed in TBST and incubated with HRP-conjugated secondary antibodies (Santa Cruz Biotechnology, sc-516102; sc-2357) for 1 h at room temperature. Bands were developed using Amersham ECL (Cytiva, RPN2106), imaged on a LAS-4000 system (Fujifilm) and quantified using ImageJ. Primary antibodies were COL1 (Abcam, ab6308), TNMD (Abcam, ab203676), SCX (Santa Cruz, sc-518082), TNC (Abcam, ab108930), MMP-1 (Santa Cruz, sc-58377) and β-actin (Santa Cruz, sc-47778).

### Scratch wound-healing assay in human tenocytes

Human tenocytes were seeded in 12-well plates at 1 × 10^5^ cells/well in DMEM supplemented with 10% FBS and cultured to confluent. Cells were washed once with DPBS, and a linear scratch was generated across the center of each well using a sterile yellow pipette tip. Detached cells and debris were removed by washing with DPBS. Cells were then incubated with THP-1 macrophage CM at 10% (v/v) as described above. Phase-contrast images of the scratch area were acquired immediately after wounding (0 h) and at 48 h using an inverted microscope. Wound area was quantified using ImageJ.

### Statistical analysis

Statistical analyses were performed using GraphPad Prism (v10). Data are presented as mean ± SD. A significance threshold of *P* < 0.05 was used. The statistical tests applied to each dataset are indicated in the corresponding figure legends.

## Results

### Platelets and p-mito show distinct post-treatment tendon thickness trajectories

**Figure 2:**
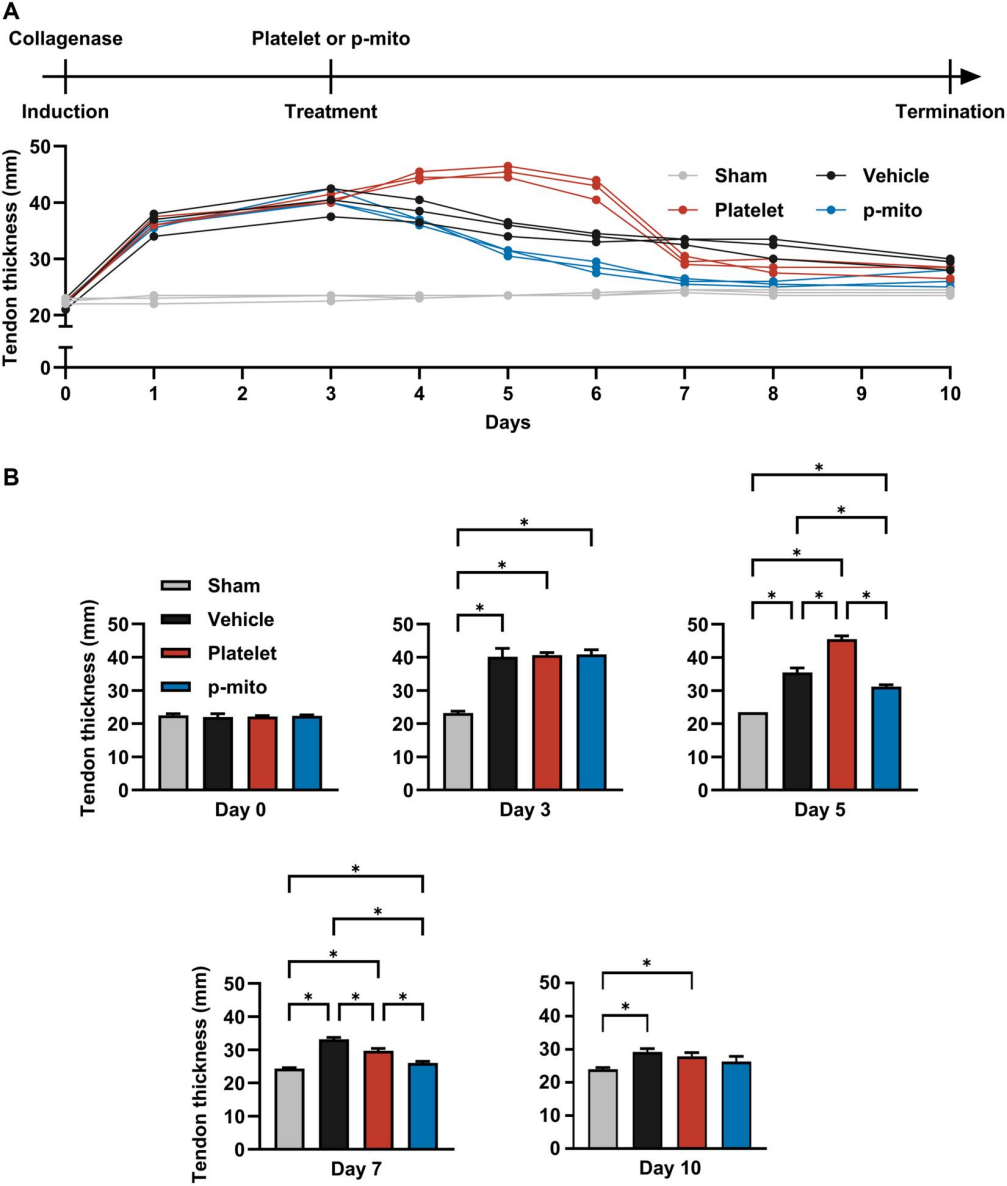
Experimental design and tendon thickness following collagenase-induced Achilles tendon inflammation in rats treated with platelets or p-mito. (**A**) Experimental timeline. Collagenase was administered to induce Achilles tendon inflammation, followed by platelets or p-mito injection on Day 3. Tendon thickness was measured serially for *N* = 3 rats/group, with left and right Achilles measurements averaged per animal. (**B**) Tendon thickness summarized by time point (Days 0, 3, 5, 7 and 10). Statistical comparisons were performed by one-way ANOVA followed by Tukey’s multiple-comparisons test at each time point using animal-level mean tendon thickness values. These analyses represent cross-sectional group comparisons at each assessed day rather than a formal repeated-measures longitudinal model.

Collagenase administration increased Achilles tendon thickness, rising through Day 3 and then declining over time in vehicle-treated animals (Figure 2A and B). To test whether platelets or p-mito altered this early course, we administered platelets or p-mito on Day 3 post-induction and monitored tendon thickness serially (*N* = 3 rats/group).

After Day 3 treatment, platelet-treated tendons were associated with an additional, transient increase in thickness, reaching the highest values around Days 4–6 relative to vehicle (Figure 2A and B). Thickness then decreased between Days 6 and 7, and platelet-treated tendons were thinner than vehicle at the Day 7 assessment (Figure 2B). In contrast, p-mito-treated tendons were associated with a consistent decline in thickness after Day 3, with values falling below vehicle by Day 5 and approaching sham levels by Days 7–10 (Figure 2A and B).

At termination (Day 10), gross inspection showed persistent swelling in the vehicle group, whereas platelet- and p-mito-treated tendons appeared closer in thickness to sham (Supplementary Figure S1). Quantitatively, vehicle-treated tendons remained thicker than sham at Day 10. Platelet-treated tendons showed an intermediate thickness above sham, and p-mito-treated tendons were not detectably different from sham at this time point (Figure 2B).

### p-mito are associated with a lower M1/M2 ratio and reduced pro-inflammatory cytokines at Day 10

**Figure 3:**
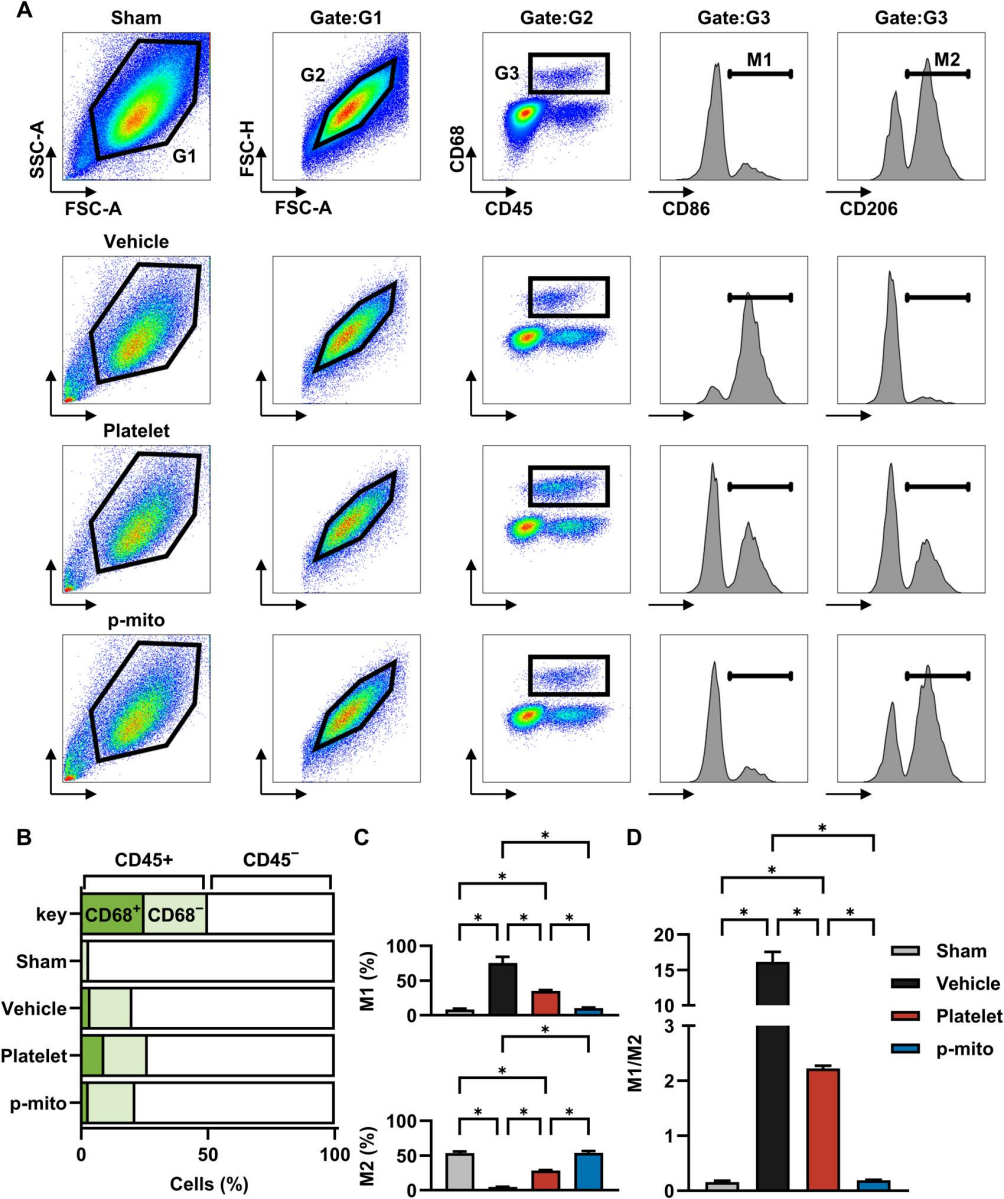
Flow cytometric analysis of macrophage subsets isolated from Achilles tendon tissue. (**A**) Representative gating strategy for identifying macrophage subsets from dissociated tendon cells. After selecting singlets (G2), CD45^+^ leukocytes and CD68^+^ macrophages were gated (G3). Within the CD45^+^CD68^+^ population, CD86^+^ cells were defined as M1-like and CD206^+^ cells as M2-like. (**B**) Frequency of CD45^+^ and CD68^+^ cells among total isolated tendon cells in each experimental group. (**C**) Proportions of CD86^+^ and CD206^+^ subsets within the CD45^+^CD68^+^ macrophage gate across groups. (**D**) M1/M2 ratio calculated from the values in (C). Statistical comparisons for (C, D) were performed using one-way ANOVA followed by Tukey’s multiple-comparisons test.

To determine whether the distinct tendon thickness trajectories after platelets or p-mito treatment were accompanied by differences in myeloid-associated profiles, we analyzed dissociated Achilles tendon cells at Day 10 post-collagenase induction (Figure 3). Singlets were gated, followed by identification of CD45^+^ leukocytes and CD68^+^ macrophages. Within the CD45^+^CD68^+^ gate, CD86^+^ cells were operationally defined as M1-like and CD206^+^ cells as M2-like (Figure 3A).

Sham tendons contained few CD45^+^ cells, whereas collagenase-treated tendons showed higher CD45^+^ frequencies (Figure 3B). Relative to vehicle- and platelet-treated tendons were associated with higher CD45^+^ and CD68^+^ frequencies at Day 10, whereas p-mito-treated tendons did not increase the CD68^+^ macrophage fraction beyond vehicle at this time point (Figure 3B).

Within the CD45^+^CD68^+^ macrophage gate, subset distributions differed across groups. Vehicle-treated tendons showed a higher CD86^+^ (M1-like) proportion with a lower CD206^+^ (M2-like) proportion (Figure 3C). Platelet-treated tendons were associated with a lower CD86^+^ proportion and a higher CD206^+^ proportion relative to vehicle (Figure 3C), with a corresponding reduction in the M1/M2 ratio (Figure 3D). p-mito-treated tendons showed a further shift in this distribution, with a lower CD86^+^ proportion, a higher CD206^+^ proportion and the lowest M1/M2 ratio among the collagenase-treated groups (Figure 3C and D).

**Figure 4:**
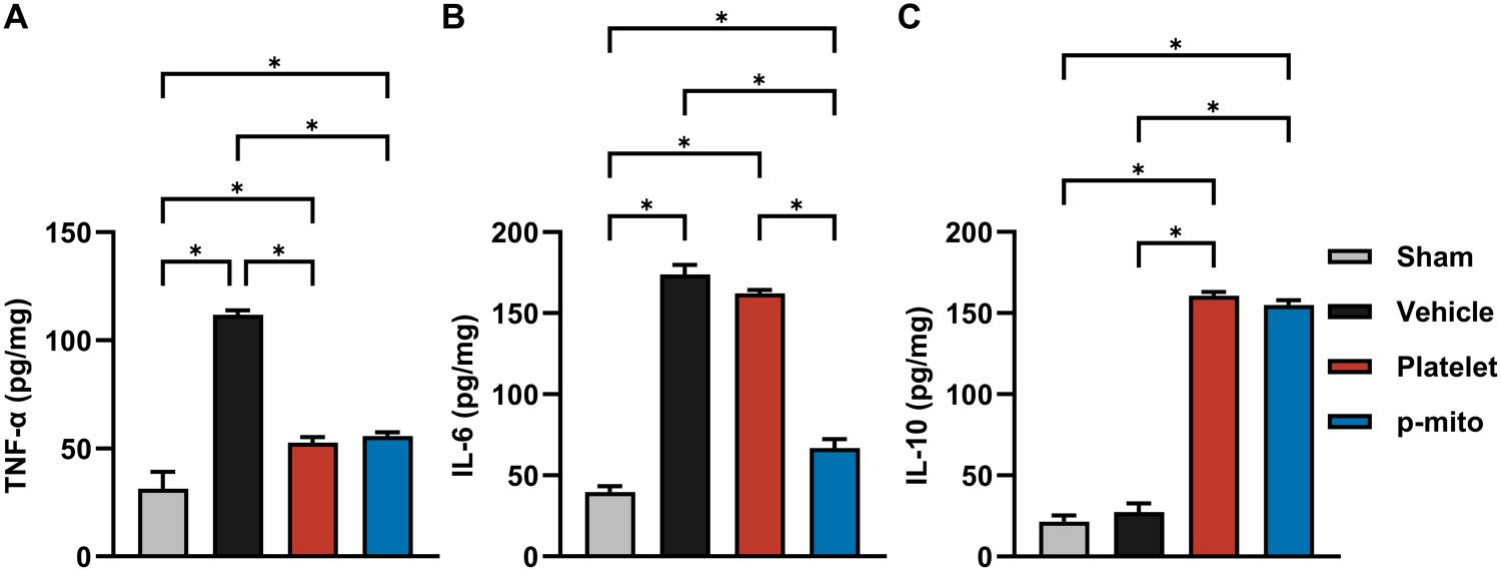
Cytokine profiling of Achilles tendon tissue homogenates by ELISA. Achilles tendon tissues were harvested at Day 10 and analyzed by ELISA to quantify (**A**) TNF-α, (**B**) IL-6 and (**C**) IL-10 in whole-tissue homogenates. Statistical comparisons among experimental groups were performed using one-way ANOVA followed by Tukey’s multiple-comparisons test.

To assess cytokine profiles at the Day 10 endpoint, we quantified TNF-α, IL-6 and IL-10 in Achilles tendon homogenates by ELISA (Figure 4). Vehicle-treated tendons showed higher TNF-α and IL-6 levels (Figure 4A and B). Platelet-treated tendons were associated with lower TNF-α at this time point but did not reduce IL-6, whereas p-mito-treated tendons were associated with lower levels of both TNF-α and IL-6 (Figure 4A and B). IL-10 remained low in the vehicle but was higher in both platelet- and p-mito-treated tendons (Figure 4C).

### p-mito were associated with higher COL1 and lower TNC than platelets at Day 10

**Figure 5:**
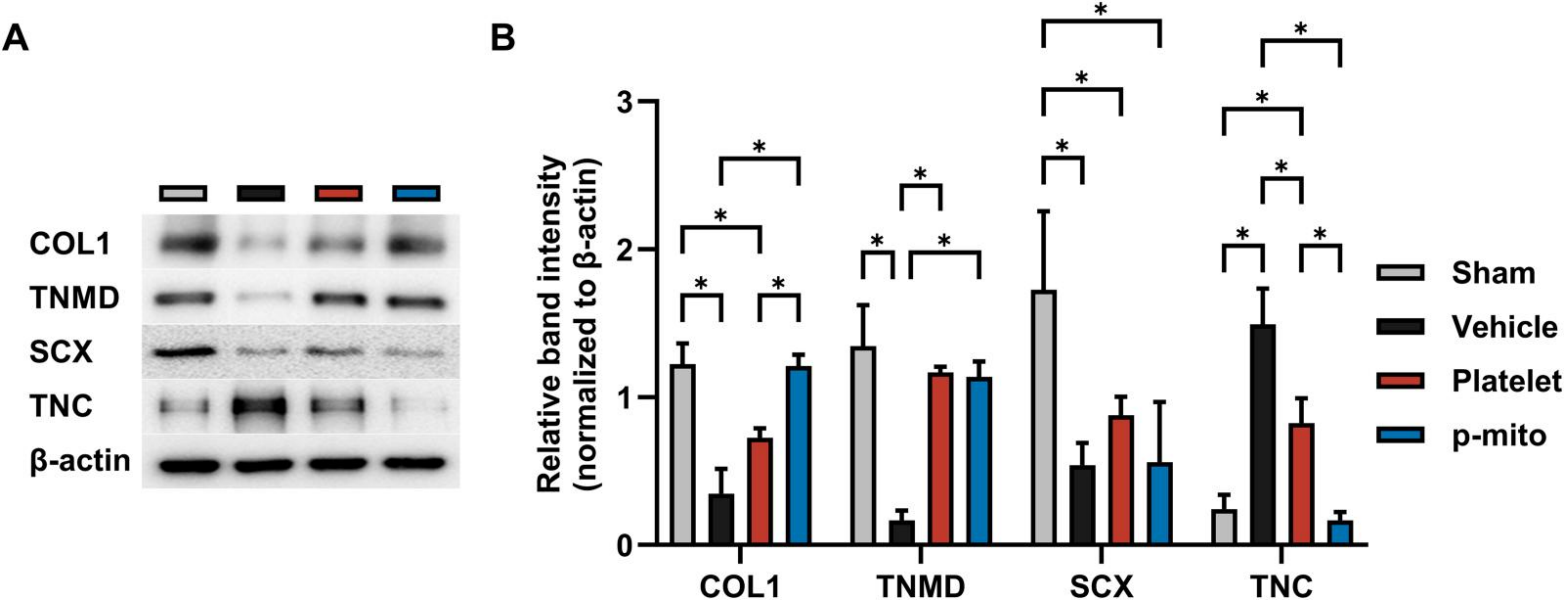
Western blot analysis of tendon-associated matrix and lineage markers in Achilles tendon tissue. Achilles tendon tissues were harvested at Day 10 and analyzed by Western blotting to assess tendon matrix and tenocyte-lineage-associated proteins. (**A**) Representative immunoblot images for COL1, TNMD, SCX, TNC and β-actin. (**B**) Densitometric quantification of band intensities normalized to β-actin. COL1, TNMD and SCX were analyzed as tendon matrix/tenocyte-lineage-associated markers, whereas TNC was analyzed as an injury-associated extracellular matrix remodeling marker. Statistical comparisons among experimental groups were performed using one-way ANOVA followed by Tukey’s multiple-comparisons test.

To assess whether the Day 10 immune-associated differences were accompanied by changes in tendon-associated proteins, we analyzed Achilles tendon homogenates by Western blot for matrix and tenocyte-lineage-associated markers (COL1, TNMD and SCX) and an injury-associated extracellular matrix remodeling marker (TNC; Figure 5A and B). At Day 10, collagenase-treated vehicle tendons showed lower COL1, TNMD and SCX and higher TNC relative to sham (Figure 5B).

Treatment groups diverged across markers. COL1 levels were similar between platelet- and vehicle-treated tendons, whereas p-mito-treated tendons showed higher COL1 than vehicle- and platelet-treated tendons. TNMD was higher in both platelet- and p-mito-treated tendons than in vehicle. In contrast, SCX remained comparable to vehicle in both treatment groups at this time point. For TNC, both platelet- and p-mito-treated tendons showed lower levels than vehicle, with p-mito-treated tendons lower than platelet-treated tendons. Together, these data are consistent with a more favorable Day 10 tendon protein profile in the p-mito-treated tendons relative to platelet-treated tendons, most notably higher COL1 and lower TNC (Figure 5B).

### Macrophage-conditioned media recapitulate platelet- versus p-mito-associated cytokine profiles and tenocyte marker responses

**Figure 6:**
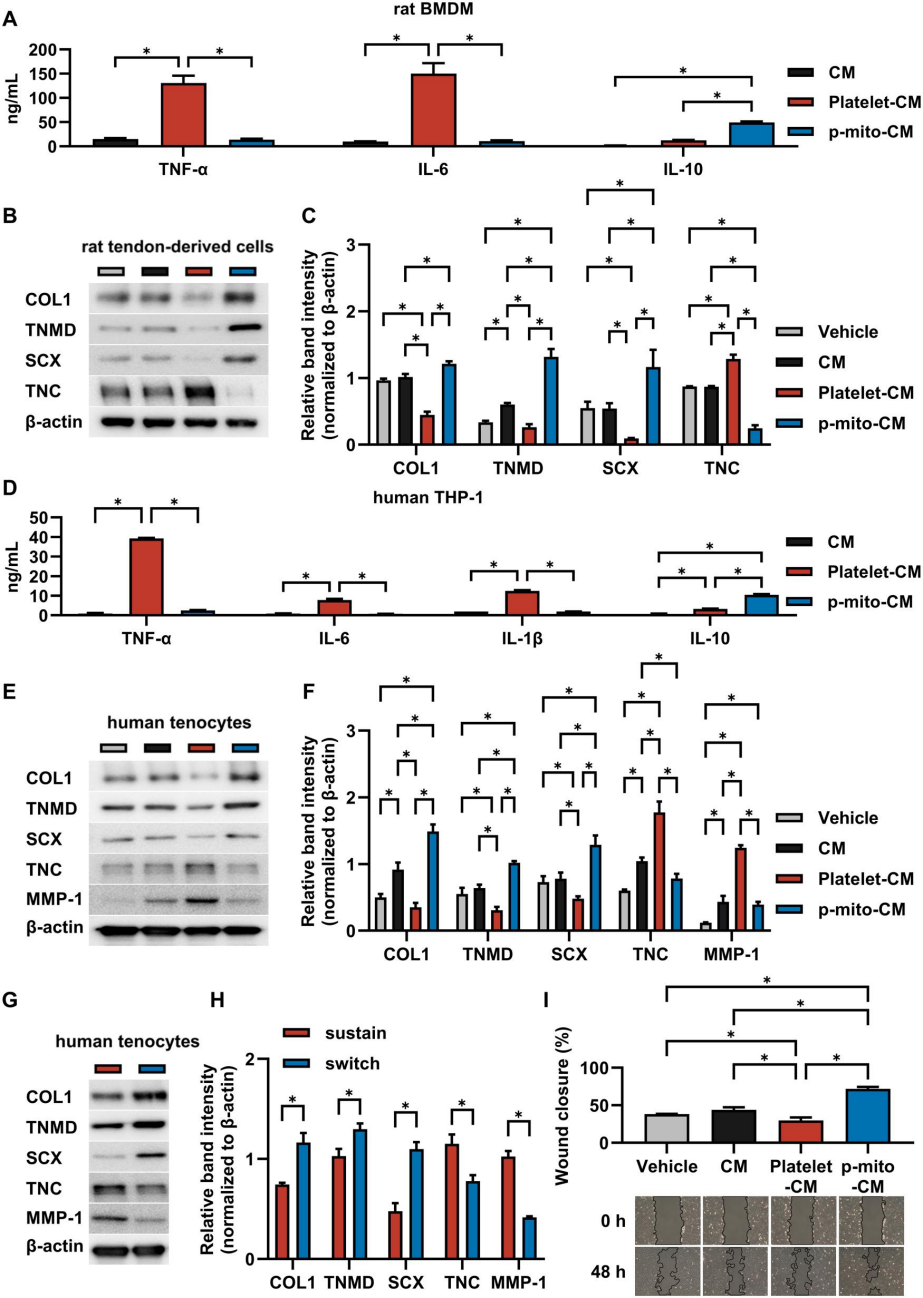
Macrophage-conditioned media model from platelet- or p-mito-associated inflammatory programs and downstream tenocyte responses *in vitro*. Macrophages were treated with platelets or p-mito for 48 h, and the resulting conditioned media (CM) were applied to tendon cells/tenocytes. Vehicle indicates culture medium only (no platelet- or p-mito-CM). CM denotes conditioned media collected from BMDM (bone marrow-derived macrophages) or THP-1 macrophages. (**A**) ELISA quantification of cytokines in CM harvested from rat BMDM treated with platelets or p-mito. TNF-α and IL-6 were analyzed as pro-inflammatory cytokines, and IL-10 as an anti-inflammatory cytokine. (**B**) Representative Western blot images of rat tendon-derived cells treated with the BMDM-CM. Immunoblots include COL1, TNMD, SCX, TNC and β-actin. (**C**) Densitometric quantification of band intensities from (B), normalized to β-actin. (**D**) ELISA quantification of cytokines in CM harvested from human THP-1 macrophages treated with platelets or p-mito. TNF-α, IL-6 and IL-1β were analyzed as pro-inflammatory cytokines and IL-10 as an anti-inflammatory cytokine. (**E**) Representative Western blot images of human tenocytes treated with the THP-1-CM. Immunoblots include COL1, TNMD, SCX, TNC, MMP-1 and β-actin. (**F**) Densitometric quantification of band intensities from (E), normalized to β-actin. (**G**) Experimental scheme comparing sustained versus switched CM exposure in human tenocytes: continuous treatment with platelet-CM for 48 h (sustain) or platelet-CM for 24 h then switched to p-mito-CM for 24 h (switch). Representative Western blot images are shown. (**H**) Densitometric quantification of band intensities from (G), normalized to β-actin. (**I**) Scratch wound-healing assay in human tenocytes. A linear scratch was introduced to form an *in vitro* wound, cells were treated with the THP-1-CM, and wound closure was assessed over time. All statistical comparisons were performed using one-way ANOVA followed by Tukey’s multiple-comparisons test.

To test whether macrophage-derived soluble factors are sufficient to reproduce platelet- versus p-mito-associated tenocyte responses, we used a conditioned media (CM) framework in rat and human systems (Figure 6). BMDMs or THP-1-derived macrophages were exposed to platelets or p-mito for 48 h, CM was collected, and tendon cells/tenocytes were treated with the resulting CM.

In rat BMDMs, platelet exposure yielded CM with higher TNF-α and IL-6, whereas p-mito exposure was associated with higher IL-10 in the CM (Figure 6A). When applied to rat tendon-derived cells, platelet-CM was associated with lower COL1, TNMD and SCX and higher TNC relative to control macrophage CM, whereas p-mito-CM showed the opposite pattern, with higher COL1/TNMD/SCX and lower TNC (Figure 6B and C).

We next assessed whether similar patterns were observed in human cells. In THP-1 macrophage CM, platelet treatment increased TNF-α, IL-6, IL-1β and IL-10, whereas p-mito treatment yielded CM characterized by higher IL-10 with comparatively lower pro-inflammatory cytokines (Figure 6D). Consistent with the rat system, platelet-CM applied to human tenocytes was associated with lower COL1, TNMD and SCX and higher TNC and MMP-1, whereas p-mito-CM was associated with higher COL1/TNMD/SCX and lower TNC (Figure 6E and F).

To assess whether a platelet-CM-induced tenocyte protein pattern could be altered by subsequent exposure to p-mito-CM, we compared sustained platelet-CM exposure with a switch paradigm (platelet-CM for 24 h followed by p-mito-CM for 24 h) (Figure 6G). Switching to p-mito-CM shifted protein levels toward higher COL1, TNMD and SCX and lower TNC and MMP-1 compared with sustained platelet-CM exposure (Figure 6G and H). In a scratch wound-healing assay, platelet-CM was associated with slower wound closure, whereas p-mito-CM was associated with faster closure over time (Figure 6I).

## Discussion

In this study, we asked whether a p-mito preparation yields responses distinct from a platelet-based preparation when administered during the early inflammatory phase of tendon injury. This question was motivated by the clinical context in which PRP can be accompanied by a transient inflammatory flare and by evidence that mitochondria may contribute to platelet-associated bioactivity [21, 30–33]. Building on our prior observation that platelets and p-mito are associated with divergent macrophage polarization signatures, we hypothesized that isolating p-mito could preserve immunomodulatory activity while reducing exposure to other platelet-associated components [22]. In a collagenase-induced Achilles tendon injury model in rats with treatment on Day 3, p-mito administration was associated with a more rapid post-treatment decline in tendon thickness, whereas platelets were associated with a transient increase followed by a later decline (Figure 2). At the Day 10 endpoint, p-mito were associated with a lower operational M1/M2 ratio within the CD45^+^CD68^+^ macrophage gate (Figure 3), lower TNF-α and IL-6 with higher IL-10 in whole-tissue homogenates (Figure 4), and a tendon protein profile characterized by higher COL1 and lower TNC relative to platelets (Figure 5). In complementary conditioned media experiments in rat and human macrophage-tenocyte systems, platelet- and p-mito-exposed macrophages generated distinct cytokine profiles accompanied by corresponding tenocyte protein patterns (Figure 6), consistent with the possibility that macrophage-derived soluble factors contribute to between-group differences observed *in vivo*.

To contextualize our emphasis on early monocyte/macrophage-lineage responses, we reanalyzed a publicly available multi-time point bulk RNA-seq tendon injury dataset (GSE288444). Because intra-tendinous delivery of platelet- or p-mito-based preparations could plausibly influence multiple resident and infiltrating cell populations, we used this reanalysis to identify immune-associated signals with prominent temporal variation soon after injury [34–37]. Immune deconvolution with CIBERSORTx yielded inferred immune cell fractions that differed across Days 0–7, including temporal variation in monocyte/macrophage-associated fractions during the first week (Figure 1A). Differential expression analyses indicated broad transcriptional remodeling at Days 1, 3 and 7 relative to Day 0, and curated M1-like and M2-like gene-set summaries showed time-dependent patterns over the same interval (Figure 1C and D). Because these outputs reflect bulk tissue averaging and model-based inference, they do not assign signals to specific cell types or establish macrophage state transitions [38–40]. We therefore treat them as descriptive context supporting the view that myeloid-associated signatures vary across early time points.

We administered platelets or p-mito on Day 3 to interrogate an early inflammatory window rather than a chronic tendinopathy stage. Although many clinical tendon conditions are treated in subacute or chronic phases, the collagenase model used here reflects an acute inflammatory injury in which swelling and leukocyte infiltration are prominent and may influence subsequent remodeling [41–44]. In our longitudinal thickness measurements, Day 3 corresponded to the maximum thickness in vehicle-treated animals, providing a pragmatic baseline for evaluating post-treatment changes. This timing is consistent with our objective to test whether platelet- versus p-mito-associated immunomodulatory tendencies translate *in vivo* when delivered during the early inflammatory phase, with emphasis on resolution-associated endpoints rather than late-stage functional outcomes.

Serial tendon thickness provided a longitudinal, nonspecific readout of the gross inflammatory course following collagenase induction (Figure 2). Tendon thickness increased through Day 3 and then declined over time in vehicle-treated animals, consistent with partial resolution of the acute response [23, 25, 45]. Relative to the vehicle, platelet administration on Day 3 was associated with an additional transient increase in thickness over Days 4–6, followed by a decline by later assessments. In contrast, p-mito administration was associated with a more immediate and sustained reduction in thickness after Day 3, falling below vehicle by Day 5 and approaching sham levels by Days 7–10. These trajectories are consistent with the possibility that p-mito limits the magnitude or duration of early post-induction swelling, whereas platelet exposure may transiently augment this thickness readout before subsequent improvement. Because thickness is an indirect surrogate and was assessed in a small cohort (*N* = 3 rats/group), these findings should be interpreted cautiously. Nonetheless, the longitudinal measurements support the conclusion that p-mito are associated with a faster post-treatment decline in tendon thickness than platelets in this model.

At the Day 10 endpoint, flow cytometry showed between-group differences in leukocyte- and macrophage-associated fractions in collagenase-treated tendons relative to sham (Figure 3). Collagenase-treated groups exhibited higher CD45^+^ frequencies than sham, consistent with increased leukocyte content at this endpoint. Because multiple immune lineages contribute to the CD45^+^ compartment, we focused downstream analyses on CD45^+^CD68^+^ cells and used CD86 and CD206 as operational markers of M1-like and M2-like subsets [46–48]. Within this framework, platelet treatment was associated with higher CD45^+^ and CD68^+^ frequencies than vehicle, whereas p-mito did not increase the CD68^+^ fraction beyond vehicle and yielded the lowest operational M1/M2 ratio among collagenase-treated groups. At the same endpoint, cytokines measured in tendon homogenates showed concordant differences: platelets were associated with lower TNF-α but not IL-6, whereas p-mito were associated with lower TNF-α and IL-6 together with higher IL-10 (Figure 4). Because these cytokines were measured in whole-tissue homogenates, the data do not identify producing cell types or establish causality. Nonetheless, the Day 10 profiles are consistent with platelet-treated tendons retaining a relatively less resolved pro-inflammatory cytokine profile than p-mito-treated tendons under the conditions tested.

We next examined tendon-associated protein markers at Day 10 to relate the immune-associated endpoint profiles to protein-level tissue readouts (Figure 5). COL1 and TNC provide complementary marker-level information relevant to tendon injury and remodeling, with COL1 representing the predominant structural collagen in the tendon matrix and TNC commonly elevated in injury-associated remodeling contexts [49–51]. Relative to sham, vehicle-treated tendons exhibited lower COL1 and higher TNC, whereas both treatment groups showed lower TNC than vehicle, with p-mito lower than platelets. At this endpoint, p-mito were associated with higher COL1 than both vehicle and platelets, whereas COL1 was similar between platelets and vehicle at this endpoint. TNMD and SCX were included as tenocyte-lineage-associated markers [52, 53]. TNMD was higher in both platelet- and p-mito-treated tendons than in vehicle, whereas SCX remained comparable to vehicle. Together, these Day 10 comparisons are consistent with partial normalization of selected markers with platelet treatment and a more pronounced change in COL1 and TNC with p-mito under the conditions tested. Whether these differences reflect distinct remodeling kinetics or converge at later time points will require additional follow-up with extended sampling and functional readouts.

To assess whether macrophage-derived soluble factors are sufficient, under simplified conditions, to reproduce platelet- versus p-mito-associated tenocyte responses under simplified conditions, we used a macrophage CM framework in rat and human systems (Figure 6A−F). Based on our prior observations that platelets and p-mito are associated with divergent macrophage polarization signatures, we reasoned that differences in macrophage-derived cytokine environments could translate into measurable changes in tenocyte-associated protein readouts [22, 54–57]. Consistent with this premise, CM generated after platelet exposure exhibited a relatively more pro-inflammatory cytokine profile and was associated with lower COL1/TNMD/SCX and higher TNC (and MMP-1 in human tenocytes), whereas CM generated after p-mito exposure was characterized by higher IL-10 with comparatively lower pro-inflammatory cytokines and was associated with the opposite tenocyte marker pattern. A CM switch paradigm further showed that replacing platelet-CM with p-mito-CM shifted tenocyte protein levels toward higher COL1/TNMD/SCX and lower TNC/MMP-1 relative to sustained platelet-CM exposure (Figure 6G and H). In parallel, the scratch assay indicated differences in wound-closure kinetics across CM conditions (Figure 6I). Although this reductionist framework does not identify the mediators responsible for these effects or establish *in vivo* causality, it supports soluble factors present in platelet- or p-mito-exposed macrophage cultures as a plausible contributor to platelet- versus p-mito-associated differences across assays. We focused here on cytokines as an initial soluble-factor readout; however, the conditioned-media effects are unlikely to be explained by cytokines alone. Other mediator classes, including lipid mediators, reactive oxygen/nitrogen species and growth factors, may also contribute to the observed tenocyte responses [58–60]. Conceptually, these findings suggest that early tendon remodeling may be shaped not only by the growth factor content of an injected biologic but also by its immunologic and mitochondria-associated properties. In this sense, p-mito may represent a mechanistically distinct acellular alternative to platelet-based preparations, targeting the early inflammatory phase, with the potential to limit excessive inflammatory priming while preserving resolution-associated repair signals [22]. Translationally, the present study provides early preclinical rationale for stepwise evaluation of p-mito in tendon repair, with future studies needed to establish matched-species efficacy, optimize dose and treatment timing, compare p-mito directly with clinically standardized PRP formulations and incorporate safety, biodistribution, histological and biomechanical outcome assessments before clinical application.

Mechanistically, the basis for the platelet–p-mito divergence observed here is likely multifactorial. Our prior work showed that platelet-derived and p-mito are associated with M2-linked macrophage polarization signatures, and that this effect depends on mitochondrial integrity and function, supporting the view that transferred mitochondria act as biologically active organelles rather than inert particles [22]. One plausible explanation is that p-mito support oxidative metabolism and bioenergetic competence in exposed macrophages, thereby favoring programs associated with inflammatory resolution [61]. A second, nonexclusive possibility is that p-mito influence macrophage behavior through organelle-derived signaling linked to mitochondrial functional state, which could in turn contribute to changes in the soluble milieu captured in the conditioned-media experiments [62, 63]. However, the present study did not directly distinguish between metabolic support, organelle-derived signaling or specific downstream pathway activation in macrophages within the tendon microenvironment. Accordingly, pathway-level mechanisms, including possible AMPK- or PKA-associated signaling, remain hypotheses for future study rather than mechanisms demonstrated here [64, 65].

We recognize several limitations of the present study and consider them informative for guiding subsequent work. First, the *in vivo* thickness measurements were performed in a small cohort (*N* = 3 rats/group), and therefore the observed differences should be interpreted as preliminary and hypothesis-generating rather than definitive. In addition, although serial tendon-thickness values were compared across groups at each time point, these analyses were conducted as cross-sectional comparisons of animal-level mean values and did not model within-animal longitudinal correlation across time. Accordingly, the serial thickness results should be interpreted with appropriate caution, and future studies using larger cohorts and formal repeated-measures or mixed-effects designs will be required to strengthen inference regarding treatment-related temporal patterns.

Second, immune-marker distributions, cytokines and tendon protein markers were assessed at a single terminal time point (Day 10) rather than longitudinally, precluding temporal mapping of how early thickness dynamics relate to later immune-associated and tissue-level readouts. Third, the study endpoints were restricted to tendon thickness, flow-cytometric markers, tissue-homogenate cytokines and selected tendon-associated protein readouts. Although the present data support a distinct early inflammatory and tendon-marker profile associated with p-mito relative to the platelet preparation used here, they do not by themselves establish improved tendon structure, collagen organization, mechanical performance or long-term functional recovery. Because the primary aim of this study was to compare early-phase post-treatment responses in an inflamed tendon microenvironment, our analyses were focused on serial tendon thickness and Day 10 macrophage-associated, cytokine and tendon-marker readouts. In addition, serial tendon-thickness assessment required repeated skin incision and tendon exposure for caliper-based measurement, and the measurement procedure itself may therefore have influenced the local inflammatory environment [8, 66]. Although this potential procedural effect was shared across all groups and thus helps preserve internal between-group comparability, it remains an important limitation of the longitudinal serial thickness analysis. Therefore, the present findings should be interpreted as evidence of differential early remodeling-associated responses rather than direct proof of improved tendon repair outcomes. Future studies incorporating histology, collagen organization analysis, biomechanical testing, extended follow-up and less invasive longitudinal imaging approaches such as ultrasound or MRI will be required to determine whether these early differences translate into meaningful tissue-level recovery [67–69].

Fourth, the platelet comparator used here was a platelet-based preparation and should not be interpreted as equivalent to a clinically formulated PRP product, which may differ in biological composition and clinical behavior [70–72]. Finally, the *in vivo* experiments used human-derived platelet and p-mito preparations in a rat model, and xenogeneic immune effects therefore cannot be excluded. Accordingly, some of the observed inflammatory differences may have been influenced not only by platelet- versus p-mito-specific biological properties but also by species-mismatch effects. This limitation should be considered when interpreting the *in vivo* immune readouts. Future studies using species-matched preparations will be important to determine whether the directional differences observed here are maintained in a fully species-consistent setting [73–77]. Together, these considerations indicate the value of future studies employing larger cohorts, multi-time-point sampling, expanded structural and functional endpoints and matched-species preparations to strengthen inference regarding the timing and biological significance of the observed differences.

## Conclusion

In summary, in a rat collagenase-induced Achilles tendon injury model with treatment delivered on Day 3, p-mito were associated with a more rapid post-treatment decline in tendon thickness and with Day 10 immune-marker, cytokine and tendon protein profiles that differed from those observed with a platelet-based preparation. Within the scope of the endpoints assessed here, these findings are consistent with the possibility that a mitochondria-focused preparation may support earlier resolution-associated immune and tendon-marker patterns compared with a platelet-based preparation under matched conditions. Determining whether these endpoint differences translate into improved structural organization or functional recovery will require extended follow-up with additional time points and direct mechanical and behavioral outcomes.

## Supplementary Material

rbag127_Supplementary_Data
