# Supplementary material for: Comparative effects of platelets and plasma-derived mitochondria in early-phase Achilles tendon inflammation
Source: Regen Biomater. 2026 Jun 13;13:rbag127. doi: 10.1093/rb/rbag127 (PMC13329078; doi:10.1093/rb/rbag127)
Supplement: rbag127_Supplementary_Data [file rbag127_supplementary_data.zip › Supplementary File_SH Kim et al.docx]

**[Supplementary File]**

**Comparative effects of platelets and plasma-derived mitochondria in early-phase Achilles tendon inflammation**

Seong-Hoon Kim^1^, Eun-Seo Back^2^, Mina Lim^1^, Mi Jin Kim^3^, Chang-Koo Yun^3,4,^*, Yong-Soo Choi^1,2,3,5,^**

^1^Department of Bio-convergence Science, Graduate School, CHA University, Seongnam 13488, Republic of Korea

^2^Department of Life Sciences, Graduate School, CHA University, Seongnam 13488, Republic of Korea

^3^Department of Life Sciences, CHA University, Seongnam 13488, Republic of Korea

^4^The Institute of AI-driven Industrial Biotechnology, Inha University, Incheon 22212, Republic of Korea

^5^Department of Medicinal Biosciences and Bioengineering, Inha University, Incheon 22212, Republic of Korea

Corresponding authors:

* ckyun@cha.ac.kr (C.K. Yun)

** yschoi93@inha.ac.kr (Y.-S. Choi)

**
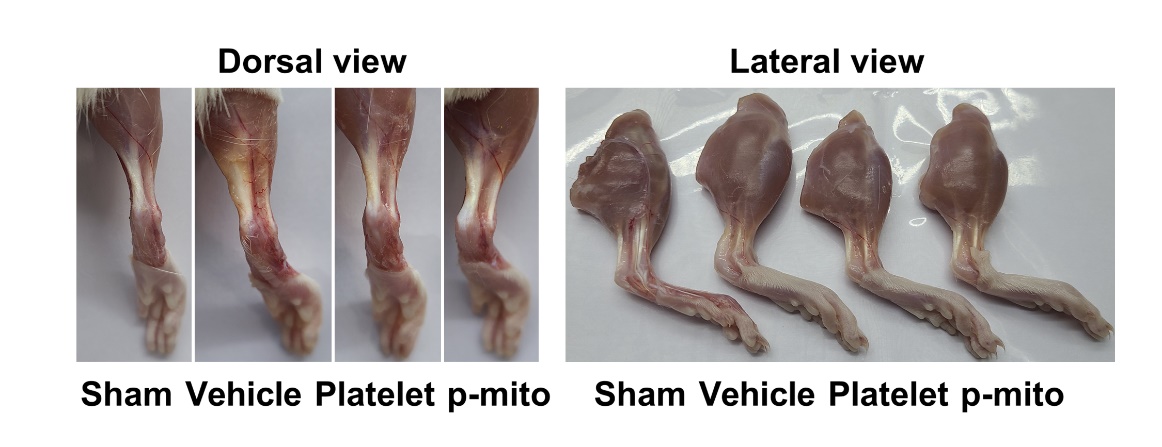
**

**Supplementary Figure S1. Representative gross morphology at termination (Day 10) after skin removal, shown in dorsal and lateral views.**
